# Potential targets for ovarian clear cell carcinoma: a review of updates and future perspectives

**DOI:** 10.1186/s12935-015-0267-0

**Published:** 2015-12-15

**Authors:** Shinya Matsuzaki, Kiyoshi Yoshino, Yutaka Ueda, Satoko Matsuzaki, Mamoru Kakuda, Akiko Okazawa, Tomomi Egawa-Takata, Eiji Kobayashi, Tadashi Kimura

**Affiliations:** Department of Obstetrics and Gynecology, Osaka University Graduate School of Medicine, 2-2 Yamadaoka, Suita, Osaka 565-0871 Japan

**Keywords:** Clear cell carcinoma, Platinum resistance, Annexin A4, Target-based therapies, Ovarian cancer

## Abstract

Advances in surgical and medical treatments for ovarian cancer have improved prognoses. Platinum drugs in particular are pivotal for the medical treatment of ovarian cancer. However, previous studies have revealed that some histological subtypes, such as clear cell carcinoma, are resistant to medical treatment, including that with platinum drugs. Consequently, the clinical prognosis of advanced clear cell carcinoma is remarkably inferior, primarily because of its chemoresistant behavior. The prevalence of clear cell carcinoma is approximately 5 % in the West, but in Japan, its prevalence is particularly high, at approximately 25 %. Current medical treatments for advanced clear cell carcinoma are difficult to administer, and they have poor efficacy, warranting the development of novel target-based therapies. In this review, we describe medical treatments for clear cell carcinoma and discuss future prospects for therapy. In particular, we focus on the mechanism of platinum resistance in clear cell carcinoma, including the role of annexin A4, one of the most investigated factors of platinum resistance, as well as the mutant genes and overexpressed proteins such as VEGF, PI3K/AKT/mTOR signaling pathway, *ARID1A*, hepatocyte nuclear factor-1β, *ZNF217*. We also review targeted molecular therapeutics for epithelial ovarian cancer and discuss their role in clear cell carcinoma treatment. We review the drugs targeting angiogenesis (bevacizumab, sorafenib, and pazopanib), growth factors (gefitinib, erlotinib, lapatinib, trastuzumab, and AMG479), and signaling pathways (temsirolimus, dasatinib, and imatinib), and other drugs (oregovomab, volociximab, and iniparib). This current review summarizes and discusses the clinical significance of these factors in ovarian clear cell carcinoma as well as their potential mechanisms of action. It may provide new integrative understanding for future studies on their exact role in ovarian clear cell carcinoma.

## Background

Ovarian cancer has the highest mortality among gynecological cancers and is associated with 4.2 % of all cancer-related deaths in women [1]. The four major histological subtypes include serous adenocarcinoma (SAC), clear cell carcinoma (CCC), endometrioid adenocarcinoma, and mucinous adenocarcinoma. Although the underlying reason remains unknown, CCC prevalence varies with race, with an estimated prevalence of 1–12 % in Europe and North America [2] and 15–25 % in Japan. Thus, CCC is the second most common histological subtype of epithelial ovarian cancer (EOC) in Japan [2, 3]. Over the past decade, advances in surgical and chemotherapeutic management have improved progression-free survival (PFS) and overall survival (OS) rates.

Platinum and taxane agents are typically included in standard intravenous regimens administered to women requiring first-line chemotherapy for ovarian cancer [4], and high response rates (60–80 %) have been shown with them. Although these chemotherapies have improved PFS and OS in ovarian cancer, some histological subtypes have shown low response rates. Moreover, standard chemotherapy using paclitaxel and carboplatin has exhibited an approximately 70 % response rate in the treatment of SAC, the most common ovarian carcinoma subtype, but only 18–56 % for CCC [3, 5, 6].

CCC tumors tend to present at earlier stages, with 47–81 % diagnosed at stage I or II, and showing a similar prognosis to SACs [7]. However, advanced CCC (i.e., FIGO stage III or IV) has a poorer prognosis, and available treatments are less effective owing to its resistance toward chemotherapeutic agents. Accordingly, in comparison with advanced SAC, the clinical prognosis for advanced CCC is remarkably inferior, primarily because of its chemoresistant behavior [8–10].

As an alternative to platinum drugs, irinotecan has been shown to be a promising candidate for the treatment of CCC in retrospective studies [11, 12] and a randomized phase II trial [13]. However, the combination therapy of irinotecan plus cisplatin (CPT-P) failed to show efficacy. A recent randomized phase III trial of paclitaxel plus carboplatin (TC) therapy versus irinotecan plus cisplatin (CPT-P) was conducted by the Japanese Gynecologic Oncology Group (JGOG 3017). This trial was the first CCC-specific international clinical trial. With a 44.3-month median follow-up, the 2-year PFS was 73.0 % in the CPT-P arm and 77.6 % in the TC arm. The 2-year OS was 85.5 % in the CPT-P arm and 87.4 % in the TC arm. That is, there were no significant changes in PFS and OS at 2 years between the two groups.

Based on these findings, CCC is considered a highly malignant and chemoresistant type of ovarian cancer, and conventional chemotherapy is not regarded as an effective treatment. In this review, we focus on potential therapeutic molecular targets and discuss prospective treatments.

## Review

### Molecular mechanisms of platinum resistance in ovarian CCC

**Fig. 1:**
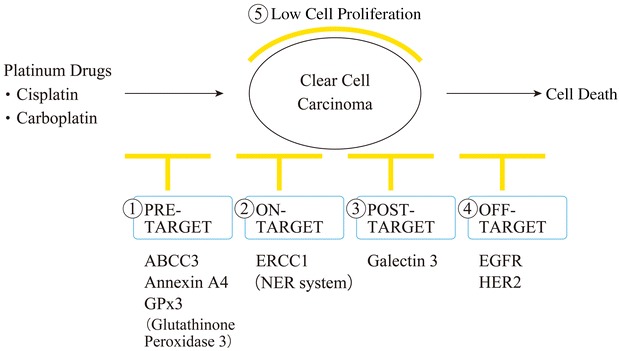
Mechanisms of platinum resistance in CCC. At present, five mechanisms of platinum resistance have been characterized in CCC, including pre-, on-, post-, and off-target mechanisms, and slow cell proliferation. *ABCC3* ATP-binding cassette, subfamily C, member 3, *ERCC1* excision repair cross-complementing rodent repair deficiency complementation group 1, *EGFR* epidermal growth factor receptor, *HER2* human epidermal growth factor receptor 2

Several mechanisms involved in platinum resistance have been proposed, including pre-, on-, post-, and off-target mechanisms as well as the speed of cell proliferation [14]. Our consideration of platinum resistance in CCC is shown in Fig. 1 and summarized in Table 1 [15–53].

**Table 1:** Summary of potential targets for platinum resistance in CCC

Category of platinum resistance	Target molecule
*Pre target*	
	*ABCC3*
Function	ABCC3 is expressed in the liver, small intestine, and colon. ABCC3 belongs to the ABCC subfamily, consisting of 13 members in mammals that are divided into three classes: multi-drug resistance proteins, sulfonylurea receptors, and the cystic fibrosis transmembrane conductance regulator [15]. ABCC3 transports monovalent bile salts (i.e., taurocholate and glycocholate) and sulfated bile salts(i.e., taurochenodeoxycholate-3-sulfate, taurolithocholate-3-sulfate) [15]
In cancer tissues	Overexpression of ABCC3, which transports chemotherapeutic agents, has been associated with paclitaxel resistance in breast cancer cell lines [16, 17] and cisplatin resistance in ovarian cancer cell lines [18]
In CCC	One study reported that ABCC3 mRNA expression in CCC was significantly higher than that in SAC [19]. However, no study to date has investigated potential mechanisms of platinum resistance in CCC
	*Annexin A4*
Function	Annexin A4 (Anx A4) is a member of the Ca^2+^-regulated and phospholipid-binding annexin protein superfamily, and is believed to be involved in exocytosis and regulation of epithelial Cl^−^ secretion [20]
In cancer tissues	Studies indicate that Anx A4 up-regulation promotes tumor progression and chemoresistance in colorectal cancer, esophageal squamous cell carcinoma, endometrial carcinoma, gastric cancer, chemoresistant lung cancer, malignant mesothelioma, renal cell carcinoma, ovarian clear cell carcinoma (CCC), cholangiocarcinoma, hepatocellular carcinoma, breast cancer, and laryngeal cancer [21]
In CCC	Enhanced Anx A4 expression was identified in both clinical samples and ovarian CCC cell lines by 2-D differential gel electrophoresis (2D-DIGE) and mass spectrometry [22]. Anx A4 confers chemoresistance to ovarian CCC cell lines [23]. Proposed mechanisms of annexin-A4-mediated chemoresistance include (1) reduction of intracellular platinum content and (2) enhancement of NF-kB transcriptional activity via interaction of Anx A4 with NF-kB. Anx A4 is one of the most investigated platinum resistance factors in CCC [20, 22–25]
	*Glutathione peroxidase 3 (GPx3)*
Function	The Glutathione Peroxidase (GPx) family is composed of eight members (GPx1–GPx8) that play roles in removing redundant reactive oxygen species (ROS) to reduce oxidative damage to host cells. The GPx3 gene is located on chromosome 5q23 and encodes a protein that accounts for nearly all GPx activities in plasma [26]
In cancer tissues	GPx3 expression has been reported in hepatocellular carcinoma, gastric cancer, acute myeloid leukemia, and clear cell renal cell carcinoma [27]. One study reported a correlation between GPx3 methylation and chemoresistance in head and neck cancer (HNC), which may serve as a potential prognostic indicator of HNC after cisplatin-based chemotherapy [28]
In CCC	Only one study to date has investigated the role of GPx3 in CCC [29]. The GPx3 gene was found to be highly expressed in CCC by DNA microarray analysis. GPx3 suppression by RNA interference increased cisplatin sensitivity by approximately 4-fold in CCC cell lines. Since GPx3 suppression increased cisplatin sensitivity of CCC cells, GPx3 may be a candidate gene associated with platinum resistance in CCC [29]
*On target*	
	*ERCC1 (NER system)*
Function	ERCC1 was the first human DNA repair gene identified by molecular cloning. The ERCC1 and ERCC4 genes encode the two subunits of ERCC1-XPF nuclease, an enzyme that plays an important role in DNA repair and maintenance of genomic stability [30]. ERCC1-XPF nuclease nicks DNA specifically at junctions between double-stranded and single-stranded DNA, when the single-strand is oriented in a 5′ to 3′ direction away from a junction. ERCC1-XPF is a core component of nucleotide excision repair and plays a role in interstrand crosslink repair, some pathways of double-strand break repair by homologous recombination and end-joining, as a backup enzyme in base excision repair, and in telomere length regulation [30]
In cancer tissues	ERCC1 is perhaps one of the most important components of the NER pathway and a key determinant of cisplatin resistance. When we consider the role that it plays in other DNA repair processes, such as recombination, it is observed that in recent years, ERCC1 has become one of the most studied prognostic factors of platinum therapy [31]. ERCC1 is reportedly a platinum-resistance factor in cancers of the ovary, lung, colorectum, and stomach [31–34]
In CCC	Although a detailed investigation was not conducted, one study detected higher ERCC1 mRNA levels in CCC specimens than other histological types of epithelial ovarian cancer [35]. Because ERCC1 was found to play an important role in platinum resistance in other cancers, further investigations into the role of ERCC1 in CCC are expected
*Post target*	
	*Galectin 3*
Function:	The galectins comprise a family of 14 members of β-galactoside-binding proteins, which are characterized by an affinity for β-galactosides and a conserved sequence in the carbohydrate recognition domain that binds to the carbohydrate portion of cell surface glycoproteins or glycolipids [36]. Galectin 3 is widely expressed in epithelial and immune cells in the gastric mucosa, colon mucosa, mammary epithelium, and prostate epithelium, as well as monocytes and macrophages. The Galectin 3 gene encodes a 31-kDa multifunctional oncogenic protein that plays a role in the regulation of cell growth, adhesion, proliferation, and apoptosis, as well as angiogenesis [36]
In cancer tissues	Galectin 3 has been implicated in many aspects of cancer progression, such as tumor cell adhesion, proliferation, differentiation, and metastasis [37], and is associated with platinum resistance in cancers of the ovary, pancreas, and prostate [38–41]. Galectin 3 has also been shown to protect cells against chemotherapy-induced apoptosis and has been implicated in the regulation of universal apoptosis commitment [37]
In CCC	Only one study to date has investigated the role of Galectin 3 in CCC [42]. Cisplatin-induced apoptosis was increased after Galectin 3 knock-down. Immunohistochemical staining showed that Galectin 3 expression in CCC was significantly more frequent than in SAC. Because p27 protein expression was decreased after Galectin 3 knock-down, the author concluded that Galectin 3 expression in CCC might contribute to decreased cell proliferation and lead to cisplatin resistance [42]
*Off target*	
	*EGFR*
Function	Epidermal growth factor receptor (EGFR) is a transmembrane glycoprotein that belongs to the receptor tyrosine kinase family of growth factor receptors and participates in important physiological process, such as cell survival, proliferation, and motility [43]
In cancer tissues	EGFR overexpression has been associated with advanced disease and poor survival of patients with cancers of the breast, lung, liver, prostate, and ovary [43]. Alterations to EGFR coding sequences are also frequently found in human cancers. Most variants with deletions in the extracellular domain are correlated with poor survival. In general these variants are constitutively active and confer a growth advantage and increased malignant potential to tumor cells [43, 44]. Overexpression or mutated EGFR is reportedly associated with platinum resistance in lung and ovarian cancer [14, 43, 45]
In CCC	A previous study reported EGFR overexpression in approximately 60 % CCC specimens and selective inhibition of the EGFR decreased growth and invasion of ovarian CCC cells [46]. Another study reported that EGFR inhibition increased cisplatin efficacy in ovarian CCC cells [47]. Together, the results of these studies suggest that EGFR is an important therapeutic target to improve platinum-resistance in CCC
	*HER2*
Function	HER2 (ErbB2) is a type I transmembrane protein that belongs to the EGFR family, which includes EGFR, ErbB1, HER1, HER3, and −4 (ErbB3 and −4) [48]. Normal HER2 function is associated with cellular differentiation, growth, development, and apoptosis via activation of tyrosine kinase activity through dimerization of HER2, with itself or other members of the EGFR family [48]
In cancer tissues	Amplification or overexpression of HER2 occurs in approximately 15–30 % of breast cancers and 10–30 % of gastric/gastroesophageal cancers, and serves as a prognostic and predictive biomarker. HER2 overexpression has also been identified in cancers of the ovary, endometrium, bladder, lung, colon, and head/neck. The introduction of HER2-directed therapies has dramatically influenced the outcome of patients with HER2-positive breast and gastric/gastroesophageal cancers; however, the results have proved disappointing in other HER2-overexpressing cancers [49]. The association between HER2 and platinum resistance in breast cancer cells [50] and the data of a systematic review suggest that triple-negative breast cancer have increased sensitivity to platinum-based chemotherapy [51]
In CCC	Amplification and overexpression of HER2 have been described in 14–42.9 % of Ovarian CCC cases [52, 53]. However, no report to date has investigated the association between HER2 and mechanisms of platinum resistance in CCC

*ABCC3* ATP-binding cassette, subfamily C, member 3, *ABC* ATP-binding cassette, *CCC* clear cell carcinoma, *SAC* serous adenocarcinoma, *GPx* glutathione peroxidase, *ERCC1* excision repair cross-complementing rodent repair deficiency complementation group 1, *NER* nucleotide excision repair, *EGFR* epidermal growth factor receptor, *HER2* human epidermal growth factor receptor 2. We summarized the factors of platinum resistance in CCC. We introduced the factors of normal function, function in cancer, and function in CCC

Pre-target mechanisms include at least two mechanisms using which cancer cells elude the cytotoxic potential of cisplatin before binding to cytoplasmic targets and DNA: (1) a reduced intracellular accumulation of cisplatin and (2) an increased sequestration of cisplatin by glutathione, metallothioneins, and other cytoplasmic scavengers with nucleophilic properties [14].

Previous studies have shown that the expression of the *ABCC3* gene is significantly greater in CCC than in SAC. Increased expression of *ABCC3* may, at least in part, be associated with the chemoresistant phenotype of CCC [19]. Moreover, *AnxA4* overexpression reportedly stimulates efflux of platinum drugs and induces platinum resistance [20, 54, 55]. Increased sequestration of platinum agents in CCC has also been reported, with significantly increased glutathione concentrations in cell lines after cisplatin exposure [56]. Furthermore, a study of gene expression showed that glutathione peroxidase 3, glutaredoxin, and superoxide dismutase 2 were highly expressed in CCC tumors and that elevated levels of these proteins may render the tumors more resistant to chemotherapy [57].

The on-target mechanism involves repair of adducts at an increased pace and/or the ability to tolerate unrepaired DNA lesions, reflecting activity of a particular class of DNA polymerases [14]. The majority of cisplatin lesions are removed from DNA by the nucleotide excision repair (NER) system [14, 58]. One study revealed higher mRNA expression of *ERCC1* and *XPB* genes in CCC. These genes are involved in the NER pathway of EOC and are more prevalent in CCC than in other histological subtypes of EOC [35]. This phenomenon may be related to de novo drug resistance against chemotherapeutic agents in CCC.

Post-target resistance to cisplatin may follow several alterations, including defects in signal transduction pathways and issues with cell death machinery [14]. In this mechanism, galectin-3 is associated with CCC platinum resistance, and suppression of galectin-3 reportedly leads to *cis*-diamminedichloroplatinum-induced apoptosis via decreases in p27 protein expression [42].

Regarding the off-target mechanism, accumulating evidence suggests that the cisplatin-resistant phenotype can also be maintained by alterations in signaling pathways that are not directly engaged by cisplatin, and yet, these compensate for cisplatin-induced lethal signals [14]. Epidermal growth factor receptor (EGFR) and human epidermal growth factor receptor 2 (HER2) are cell-surface receptor tyrosine kinases that are capable of activating both mitogen-activated protein kinase and phosphatidylinositol 3-kinase (PI3K)/AKT signaling pathways. This leads to phosphorylation of BAD and Bcl-2 and results in the inhibition of chemotherapy-induced apoptosis. An immunohistochemical study reported that the expression of EGFR was detected in 61 % of CCC tumors [46]. Furthermore, HER2 was reportedly overexpressed in CCC in comparison with other major histological subtypes of EOC. In ovarian cancer, the HER2 protein was overexpressed as a consequence of gene amplification in 20–25 % of cases and predicted poor prognosis [59, 60]. Moreover, HER2 was overexpressed in 42.9 % of CCC cases, as investigated using immunohistochemistry (IHC) [61].

Slow cell proliferation is also associated with platinum resistance because cytotoxic drugs primarily target proliferating cells [5], and doubling times for CCC cells were significantly longer than those for SAC cells (61.4 vs. 29.8 h) [5].

Determining the mechanisms underlying platinum resistance in CCC is important, because no novel drugs have yet proven effective for CCC treatment. Our review revealed that annexin A4 (AnxA4) is one of the most well-investigated platinum resistance factors in CCC [20, 22–25]. A recent study showed that *AnxA4* knockout improved platinum resistance of CCC in vitro and in vivo [56], and the functional site of AnxA4 that is responsible for conferring platinum resistance has been identified. If an AnxA4 blockade drug was developed, its use in combination with platinum drugs could have therapeutic activity against CCC.

### Characteristics of ovarian CCC

The molecular features of CCC are summarized in Table 2 [22, 24, 46, 52, 53, 61–72]. One major distinguishing characteristic is its higher incidence among Asian populations, particularly among Japanese women [2, 3, 56]. The reason for this is unknown, although CCC has been associated with endometriosis and endometriosis-associated ovarian cancers in 22–70 % of younger female patients [73]. Previous studies showed that ovarian endometrioma increases the risk for ovarian cancer, and 0.72 % of all cases of ovarian endometrioma later develop neoplasms [74].

**Table 2:** Characteristics of ovarian clear cell carcinomas and potential molecular targets

Clinical features	Mutated genes and overexpressed proteins	Reference
Higher incidence among Asian women, particularly Japanese women	ARID1A is mutated in 46 % of CCC patients (loss of function)	[62, 85]
Clear cell carcinoma has a strong association with endometriosis	PIK3CA is mutated in 33 % of CCC patients (activation mutation)	[53]
Slow tumor growth, facilitating early detection	Annexin A4 is expressed in almost all CCC patients	[20, 22]
Strong resistance to platinum-based chemotherapy	mTOR is overexpressed in 80 % of CCC patients	[63]
Promising regimens with favorable and stable response for ovarian clear cell carcinoma have remained elusive	HNF-1β is expressed in almost all CCC patients	[52, 64, 71–73]
Low frequency of BRCA1/2 mutations	ZNF217 is overexpressed in 20 % of CCC patients	[65]
Low frequency of p53 mutations (15 %)	VEGF is strongly expressed in both early and advanced stages of CCC	[66]
	EGFR is overexpressed in 60 % of CCC patients	[59]
	MET is overexpressed in approximately 20 % of CCC patients	[85]
	HER2 is overexpressed in 14–42.9 % of CCC patients	[52, 53]
	PPMID expression is observed in 10 % of CCC patients	[68]
	PPP2R1A is overexpressed in 7 % of CCC patients	[52]
	KRAS is overexpressed in 5 % of CCC patients	[52]

*ARID1A* AT-rich interactive domain 1A (SWI-like) gene, *BRCA 1/2* breast cancer susceptibility gene 1/2, *EGFR* epidermal growth factor receptor, *HER2* human epidermal growth factor receptor 2, *HNF-1β* hepatocyte nuclear factor 1β, *mTOR* mammalian target of rapamycin, *PIK3CA* phosphoinositide 3-kinase catalytic-α, *PPMID* protein phosphatase magnesium-dependent, *PP2R1A* protein phosphatase 2, regulatory subunit A, *VEGF* vascular endothelial growth factor, *ZNF217* zinc finger protein 217

CCC tends to present at significantly earlier stages than other ovarian cancers, possibly owing to slow tumor growth and frequent presentation of tumors as large pelvic masses [75], and the proportion of stage I/II tumors ranges from 59 to 71 % [3].

Unlike high-grade serous EOCs, CCCs usually display a wild-type p53 and have a lower frequency of *BRCA1* and *BRCA2* mutations [2, 76, 77]. Significant differences were reported in the distribution of mutations among histological subtypes, and *TP53* mutations were reportedly present in 67 and 21 % of cases with serous and nonserous cancers, respectively [76]. Similar studies report much lower frequencies of p53 mutations (approximately 15 %) in CCC than in other EOC types [2]. Similarly, Alsop et al. reported that the frequency of *BRCA1* and *BRCA2* mutations of CCC was 6.3 and 0 %, respectively [77]. However, other recent studies have revealed that several genes/proteins are mutated and/or overexpressed in CCC, and that these proteins may serve as therapeutic targets for CCC (Table 2) [22, 24, 46, 52, 53, 61–72].

### Novel therapeutic modalities for CCC

Paclitaxel plus carboplatin combination therapy is currently the primary treatment strategy in postoperative chemotherapy. However, advanced-stage patients eventually relapse after adjuvant therapy and have a high risk of recurrence [3, 5, 6]. The mechanism underlying resistance to standard chemotherapy has been studied but remains unknown. Nonetheless, novel drugs that target specific molecular pathways are being developed to improve the outcomes of chemotherapy-resistant ovarian cancer. Moreover, studies of chemotherapy-resistant ovarian cancer therapy indicate that effective treatments for CCC are available. However, clinical data relating directly to the treatment of CCC subtypes are limited.

Clinical trials have shown that in combination with chemotherapy, targeting vascular endothelial growth factor (VEGF), VEGF receptor (VEGFR), mammalian target of rapamycin (mTOR), EGFR, and poly (ADP-ribose) polymerase elicits positive results for EOC (Table 3) [52, 61, 71, 78–83]. However, limited data are available pertaining to novel therapies for either EOC or CCC, and further studies of novel treatment strategies are required to focus on the clinical features of CCC.

**Table 3:** Examples of targeted molecular cancer therapeutics for epithelial ovarian cancer

Category	Target molecule	Agent(s)	References
*Angiogenesis*	*Vascular endothelial growth factor*	*Bevacizumab*	[71, 85]
Function	Bevacizumab is a humanized recombinant monoclonal antibody that inhibits vascular endothelial growth factor (VEGF) receptor binding		
Efficacy	A clinical trial of bevacizumab addition to standard chemotherapy treatment in newly diagnosed advanced ovarian cancer demonstrated its efficacy. Bevacizumab monotherapy is effective in the treatment of persistent, resistant, or recurrent epithelial ovarian cancer (EOC). However, it remains unknown whether bevacizumab is effective for the clinical treatment of clear cell carcinoma (CCC). Some reports have suggested its efficacy in vitro and in vivo		
	*Vascular endothelial growth factor receptor*	*Sorafenib*	[71]
Function	Sorafenib is a multikinase inhibitor of intracellular Raf kinases and cell surface kinase receptors and thereby inhibits tumor growth and angiogenesis		
Efficacy	Phase I and II studies show limited clincial benefits of sorafenib in the treatment of EOC, both as monotherapy and in combination with other drugs. No data is available regarding its use in the treatment of clear cell carcinoma (CCC). However, it is regarded as a useful therapy for patients with renal CCC. Therefore, sorafenib may be efficacious in the treatment of CCC		
	*Vascular endothelial growth factor receptor*	*Pazopanib*	[71]
Function	Pazopanib is a tyrosine kinase (multikinase) inhibitor that limits angiogensis and tumor growth by inhibiting cell surface vascular endothelial growth factor receptors (VEGFRs), platelet-derived growth factor receptors, and fibroblast growth factor receptors		
Efficacy	Pazopanib monotherapy was relatively well tolerated, with similar toxicity to that of other small-molecule oral angiogenesis inhibitors. Promising single-agent activity was demonstrated in patients with recurrent ovarian cancer. Phase II and III trials indicate that pazopanib may have a role in the treatment of some women with EOC. No data demonstrates its efficacy in the treatment of CCC. However, pazopanib is regarded as useful in the treatment of renal CCC patients		
*Growth factor*	*Epidermal growth factor receptor*	*Gefitinib, erlotinib, and lapatinib*	[78]
Function	Gefitinib is a tyrosine kinase inhibitor that inhibits numerous tyrosine kinases that are associated with transmembrane cell surface receptors on both normal and cancer cells, including the epidermal growth factor receptor (EGFR) assoicated tyrosine kinase		
Efficacy	EGFR-targeted treatment had no effect when administered as monotherapy or as an adjunct to chemotherapy. However, EGFR-targeted treatment has shown promise in combination with other chemotherapeutic agents in clinical use. Further reports are expected. No data show the effects of targeting EGFR in CCC patients		
	*Human epidermal growth factor receptor 2*	*Trastuzumab*	[79]
Function	Trastuzumab is a monoclonal antibody that binds to the extracellular domain of the human epidermal growth factor receptor 2 protein (HER 2) and mediates antibody-dependent cellular cytotoxicity by inhibiting the proliferation of cells that overexpress the HER 2 protein		
Efficacy	HER-2 gene and protein overexpression have been reported in breast cancer and are associated with an aggressive clinical course and poor prognosis. A Gynecologic Oncology Group study demonstrated that HER 2 overexpression has no predictive or prognostic value in ovarian cancer. Although trastuzumab is not useful for ovarian cancer, no studies have investigated its use in the treatment of ovarian CCC. Further studies are needed to determine the efficacy of trastuzumab in the treatment of ovarian CCC		
	*Insulin-like growth factor type I receptor*	*AMG479*	[80]
Function	A complete human monoclonal antibody against insulin-like growth factor type I receptor (IGF-1R)		
Efficacy	IGF-1R inhibition with ganitumab was well tolerated and demonstrated modest single-agent activity in unselected patients with platinum-sensitive recurrent ovarian cancer. To our knowledge, two clinical trails have been completed, although the results have nto yet been published. There are no data pertaining to its use in the treatment of CCC		
Tumor Marker	*Cancer antigen 125*	*Oregovomab*	[71]
Function	Oregovomab is a monoclonal antibody that binds to the antigen cancer antigen (CA 125)		
Efficacy	A phase III clinical trial of intravenous oregovomab as post-chemotherapy consolidation has been conducted for EOC of tubal or peritoneal origin. Oregovomab monotherapy failed to improve outcomes after first line therapy. There are no data pertaining to its use in the treatment of CCC		
*Adhesion*	*α5β1 integrin*	*Volociximab*	[81]
Function	Volociximab binds to and inhibits the activity of α5β1 integrin		
Efficacy	A phase II, multicenter, single arm, two stage study evaluated the efficacy, safety, and tolerability of weekly administration of volociximab as a single agent for the treatment of platinum-resistant, advanced EOC and primary peritoneal cancer. Volociximab was well tolerated, but there is insufficient evidence of its efficacy. There are no reports of volociximab treatments for CCC		
*Signal*	*Mammalian target of rapamycin*	*Temsirolimus*	[82]
Function	The mammalian target of rapamycin (mTOR) signaling pathway senses and integrates a variety of environmental cues to regulate growth and homeostasis. Temsirolimus is an inhibitor of the mTOR pathway		
Efficacy	Inhibitors of mTOR have shown therapeutic advantages when used in combination with other therapeutic modalities. Although clinical activity was low compared with the expected benefits, warranting further investigation. Existing data demonstrates the efficacy of targeting the mTOR pathway for CCC treatment in vitro and in vivo		
	*Src*	*Dasatinib*	[71]
Function	Elevated activity of Src tyrosine kinase is suggested to be linked to cancer progression through the promotion of other signals. Dasatinib is a BCR-ABL tyrosine kinase inhibitor. It also inhibits the Src family, c-KIT, EPHA2, and platelet-derived growth factor receptor β		
Efficacy	Dasatinib has minimal activity as a single agent in patients with recurrent EOC. There are no data pertaining to its use in the treatment of CCC		
	*c-Kit*	*Imatinib*	[71]
Function	c-Kit is a receptor tyrosine kinase type III, which binds to stem cell factor, also known as “steel factor” or “c-kit ligand”. Signaling through c-kit plays a role in cell survival, proliferation, and differentiation		
Efficacy	Some reports show dissapointing results in clinical outcomes. Few patients had sustained responses or stable disease, and treatment with imatinib did not prolong progression-free survival		
*DNA repair*	*Poly ADP ribose polymerase*	*Iniparib*	[83]
Function	Proteins of the poly ADP ribose polymerase (PARP) family are involved in several cellular processes, mainly involving DNA repair and programmed cell death. Iniparib was originally believed to act as an irreversible inhibitor of PARP1		
Efficacy	Phase II multicenter, single-arm clinical studies have been conducted to assess the efficacy and safety of carboplatin/gemcitabine in combination with iniparib in patients with platinum-sensitive or -resistant recurrent ovarian cancer. Phase III clinical trial of olaparib was initiated for patients with BRCA mutant ovarian cancer. However, low frequency of BRCA1/2 mutations in CCC were reported		

*ABL* Abelson murine leukemia, *BCR* breakpoint cluster region, *CA125* cancer antigen 125, *EGFR* epidermal growth factor receptor, *EPHA2* ephrin type-A receptor 2, *GFR* growth factor receptor, *HER2* human epidermal growth factor receptor 2, *IGF-1R* insulin like growth factor-1 receptor, *mTOR* mammalian target of rapamycin, *PARP* poly (ADP-ribose) polymerase, *PDGFR* platelet-derived growth factor receptor, *VEGF* vascular endothelial growth factor, *VEGFR* vascular endothelial growth factor receptor

#### Targeting VEGF

VEGF is an important therapeutic target in several solid tumors, including ovarian cancers, and the monoclonal antibody bevacizumab has been shown to bind to VEGF, inhibit receptor binding, and prevent the growth of tumor vasculature. Accordingly, the International Collaborative Ovarian Neoplasm Group (ICON7) and the Gynecologic Oncology Group (GOG) trials (GOG218) of bevacizumab addition to standard chemotherapy in newly diagnosed advanced ovarian cancer both reported significant improvements in PFS. The ICON7 trial specifically reported increased OS in a predefined group of patients with a high risk of disease progression [84, 85]. In addition, efficacy and safety of bevacizumab has been reported both in patients with platinum-sensitive and in those with platinum-resistant recurrent ovarian cancers [84, 85]. However, in these studies, histological subgroup analyses were not performed, and the clinical utility of VEGF as a therapeutic target for CCC has not been evaluated. Mabuchi et al. demonstrated the efficacy of bevacizumab in in vitro and in vivo CCC models, which showed that VEGF is frequently expressed and may be a promising therapeutic target for the management of CCC [66]. However, a clinical trial has not been performed. Sunitinib is another possible therapeutic option for renal cell carcinoma treatment and acts as an oral, small-molecule, multitargeted receptor tyrosine kinase inhibitor (targeting VEGFR, platelet-derived growth factor receptor, and c-Kit). However, only a few small clinical studies have reported the efficacy of sunitinib for ovarian CCC [86].

#### Targeting the PI3K/AKT/mTOR signaling pathway

PI3Ks are lipid kinases that regulate signaling pathways that are vital for neoplasia, including cell proliferation, adhesion, survival, and motility. The frequency of *PIK3CA* mutations in CCC has been estimated to be 40 %, and some studies suggest that the PI3K/AKT/mTOR pathway is a target with therapeutic potential [53]. Moreover, immunohistochemical analyses have shown that mTOR is frequently activated in CCC (86.6 %) and that mTOR inhibition by RAD001 may be an effective treatment for CCC [63]. Furthermore, Rahman et al. reported that *PIK3CA* mutations were associated with more favorable prognoses but did not predict the sensitivity of ovarian CCC cells to PI3K/AKT/mTOR inhibitors [87]. Since more than 80 % of ovarian CCC shows activation of the AKT/mTOR pathway, it is of great interest to explore the potential of mTOR inhibitors [88]. A very important GOG clinical trial is currently being conducted. The GOG-268 trial is an open-label phase II trial for newly diagnosed stage III and IV ovarian CCC to examine the activity of one of the mTOR inhibitors, temsirolimus. The primary endpoint of this trial is PFS at 12 months, and secondary endpoints include adverse events, duration of PFS and OS, and tumor response. IHC expression of components of the mTOR signaling pathway will be explored. Temsirolimus will be administered in combination with paclitaxel and carboplatin for six cycles. For the maintenance phase, temsirolimus will be administered on days 1, 8, and 15 every 3 weeks for 11 cycles. This clinical trial closed, and clinicians are awaiting the results with high expectations. Although targeting the PI3K/AKT/mTOR signaling pathway is promising, some problems remain, and there is no evidence of effective clinical management of CCCs, warranting further studies and clinical trials to prove the efficacy of PI3K/AKT/mTOR inhibition.

#### Targeting AnxA4

AnxA4 is reportedly involved in exocytosis and regulation of epithelial Cl^−^ secretion [20], and its overexpression in CCC has been shown to induce platinum resistance [22]. Accordingly, IHC analyses of AnxA4 in CCC samples showed strong staining in 30 of 43 samples but moderate staining in the remaining 13 samples [22].

Nonetheless, enhanced AnxA4 expression was recently shown to increase chemoresistance to carboplatin by contributing to extracellular efflux of the drug [22]. Recently, we demonstrated molecular mechanisms underlying AnxA4-induced promotion of platinum drug efflux [54]. Exposure of an AnxA4-overexpressing endometrial carcinoma cell line to platinum drugs caused relocalization of AnxA4 from the cytoplasm to the membrane fraction, and colocalization of P-type ATPase ATP7A (a copper and platinum transporter) to cell membranes. This colocalization promoted platinum drug efflux via ATP7A and induced the platinum resistance [54].

**Fig. 2:**
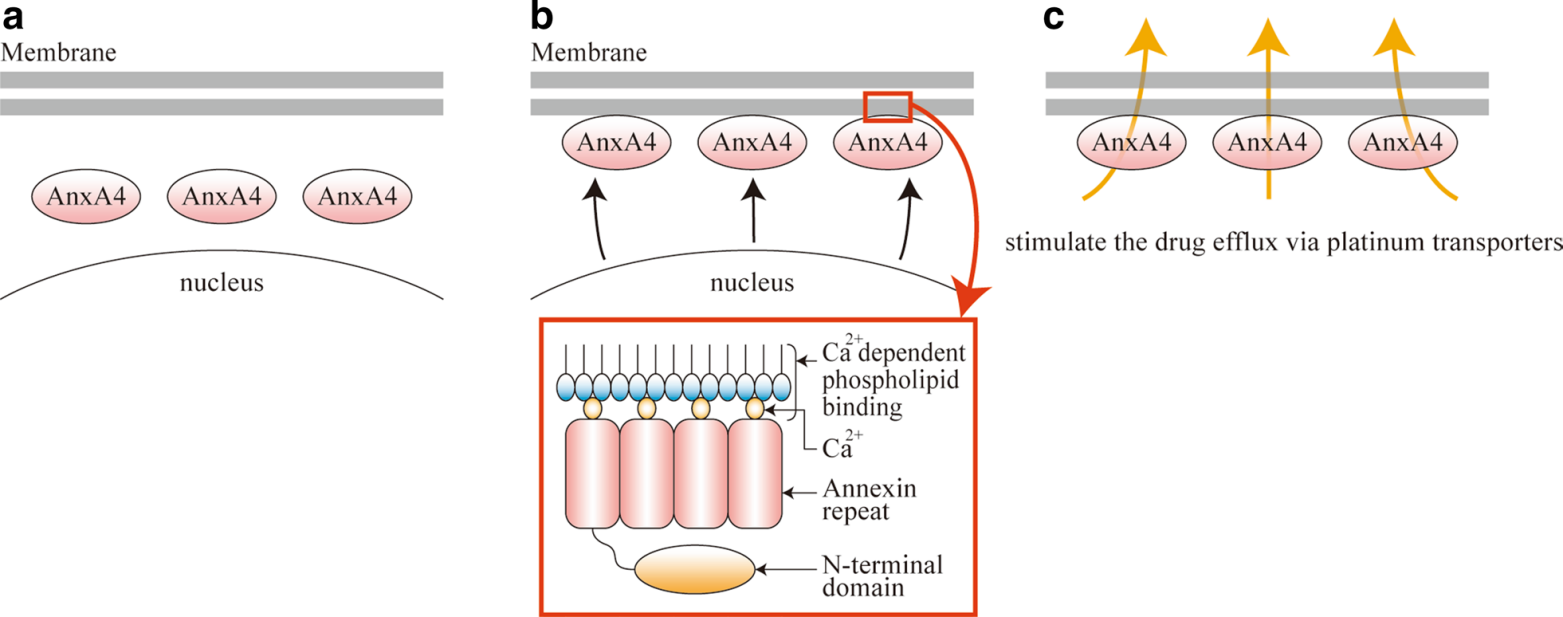
The structure of annexin A4 (AnxA4) and related mechanisms of platinum resistance. **a** No treatment. AnxA4 is localized in the cytoplasm before exposure to platinum drugs. **b** After platinum drug exposure. Exposure to platinum drugs leads to AnxA4 relocalization from the cytoplasm to the cell membrane. AnxA4 has four annexin repeats that are packed into an α-helical disk within the C-terminal polypeptide core, which contains Ca^2+^-binding sites. These Ca^2+^-binding sites are involved in platinum resistance. **c** After platinum drug exposure. AnxA4 is attached to the membrane surface through bound Ca^2+^ ions and contributes to the efflux of platinum drugs via platinum transporters

Several studies have shown that AnxA4 induces drug resistance [20], and suppression of AnxA4 expression improved platinum sensitivity of CCC in vitro and in vivo [55]. In addition, Morimoto et al. showed that annexin repeat domains and calcium-binding sites of repeated annexin sequences are required for resistance to platinum-based drugs [55]. The structure of AnxA4 and the mechanism of platinum resistance induced by AnxA4 is shown in Fig. 2. Taken together, these reports suggest the potential of AnxA4 targets for the treatment of ovarian CCC. However, no drugs have been shown to suppress AnxA4 expression. Nonetheless, in a study of the related annexin A2 (AnxA2) using chick chorioallantoic membrane assays, neutralizing antibodies significantly inhibited OV-90 cell motility and invasion in vitro and in vivo, suggesting the potential of AnxA2-neutralizing antibodies as therapeutic targets for AnxA2-overexpressing cancers [89]. Similarly, AnxA4 blockade using neutralizing antibodies might limit the platinum resistance of CCC and is currently under investigation by our research group. Potentially, drugs that inhibit the function of AnxA4 in combination with platinum drugs may offer promising therapies for the treatment of CCC.

#### Targeting the *ARID1A* gene

The AT-rich interactive domain 1A (SWI-like) gene (*ARID1A*) encodes BAF250A, which is a member of the SWI/SNF ATP-dependent chromatin remodeling complex. *ARID1A* plays an indispensable role in controlling gene expression and in tissue development and cellular differentiation [70]. Moreover, CCCs reportedly have the highest frequency of *ARID1A* mutations (43–57 %) [62].

Yamamoto et al. reported two critical associations between the AT-rich interactive domain-containing protein 1A (ARID1A) and CCC. In particular, deficiencies of ARID1A immunoreactions were evident at the stage of precursor lesions that lacked atypical cytology, indicating that the loss of ARID1A protein may occur as an early event in tumorigenesis. Moreover, loss of ARID1A protein expression is often coincident (not mutually exclusive) with *PIK3CA* mutation [90]. However, a previous study demonstrated that inactivation of ARID1A alone is insufficient for tumor initiation, suggesting that additional genetic alterations are required to drive tumorigenesis [91]. Chandler et al. also showed that the coexistent *ARID1A*-*PIK3CA* mutations promote ovarian clear cell tumorigenesis [92]. Taken together, these studies suggest that ARID1A is related to CCC tumorigenesis, although the precise mechanisms remain unknown, and no drugs have yet been shown to target ARID1A. A recent study by Bitler et al. is an important study regarding the targeting of ARID1A for ovarian CCC [93]. The study showed that *ARID1A* is mutated in over 50 % of ovarian CCCs and pharmacological inhibition of enhancer of zeste homolog 2 (EZH2) represents a novel treatment strategy for cancers involving *ARID1A* mutations. The study showed that EZH2 inhibitor selectively suppressed the growth of *ARID1A* mutated cells in vitro and in vivo via upregulating the expression of *PIK3IP1*, which negatively regulates PI3K/AKT signals. Further studies are expected to elucidate the detailed mechanisms of the cellular dysfunction caused by *ARID1A* mutations in CCCs and to expose the potential of ARID1A as a therapeutic target.

#### Targeting hepatocyte nuclear factor-1β

Hepatocyte nuclear factor-1β (HNF-1β) is a transcription activator that regulates the promoters and enhancers of genes expressed in the liver, digestive tract, pancreas, and kidneys [64]. Recent studies have reported specific expression of HNF-1β in endometriosis and CCC and suggest that early differentiation into the clear cell lineage occurs in endometriosis [64, 74]. However, the role of HNF-1β expression in ovarian clear cell tumors and endometriosis remains uncertain. Nonetheless, RNA interference has been used to decrease HNF-1β expression and reportedly led to apoptotic cell death in CCC cell lines, indicating that HNF-1β expression may be tightly linked to CCC and that it could be essential for its survival [74]. In addition, HNF-1β is reportedly expressed in almost all CCC cases [64].

Accordingly, Kajihara et al. concluded that the HNF-1β-dependent pathway may provide novel insights into the regulation of glycogen synthesis, detoxification, and resistance to anticancer agents [94]. In support of these conclusions, HNF-1β directly regulates multiple cancer-related genes, including those for dipeptidyl peptidase IV, osteopontin, tissue factor pathway inhibitor 2, AnxA4, and angiotensin-converting enzyme 2 [74, 94, 95]. Genes that are upregulated in CCC are likely direct targets of HNF-1β. However, drugs that target HNF-1β have not been developed, warranting further studies of the mechanisms by which HNF-1β regulates various genes and its association with CCC.

#### Targeting *ZNF217*

The *ZNF217* gene on human 20q13.2 encodes a transcription factor that is overexpressed in 30 % of breast tumors and in several cell lines [96]. Several studies show that overexpression of *ZNF217* in several cancers is associated with poor prognosis [96, 97]. Among these, Littlepage et al. reported that *ZNF217* overexpression promotes metastasis and resistance to chemotherapy and inhibits signaling events in vivo [96]. These authors also showed that triciribine inhibits the growth of *ZNF217*-overexpressing cells in vitro and in vivo, indicating that it is a potential target for the treatment of *ZNF217*-overexpressing cancers.

In a previous study, *ZNF217* overexpression was reported in 20.0 % of CCC cases [65]. Moreover, Rahman et al. showed that *ZNF217* gene overexpression is significantly correlated with lymph node metastasis in ovarian CCC. In comparison with small interfering RNA-treated cells without *ZNF217* overexpression, profound inhibition of cell migration and invasion was observed in cells overexpressing *ZNF217* [98], suggesting that *ZNF217* is a potential therapeutic target for CCC.

## Conclusion

As discussed above, the loss of *ARID1A* expression and/or PI3K activation is crucial for CCC tumorigenesis. Moreover, synergic effects of the loss of *ARID1A* expression and PI3K/AKT pathway activation and *ZNF217* overexpression may be related to ovarian CCC development [99], warranting further studies of these associations and assessments of their potential as co-therapeutic targets for CCC.

CCC is highly resistant to current platinum-based treatment. However, if an AnxA4 blockade drug was developed, its use in combination with platinum drugs may have therapeutic activity against CCC.

## Abbreviations

AnxA2: annexin A2
AnxA4: annexin A4
ARID1A: AT-rich interactive domain-containing protein 1A
CCC: clear cell carcinoma
CPT-P: irinotecan plus cisplatin
EGFR: epidermal growth factor receptor
EOC: epithelial ovarian cancer
EZH2: enhancer of zeste homolog 2
GOG: Gynecologic Oncology Group
HER2: human epidermal growth factor receptor 2
HNF-1β: hepatocyte nuclear factor-1β
IHC: immunohistochemistry
mTOR: mammalian target of rapamycin
NER: nucleotide excision repair
OS: overall survival
PFS: progression-free survival
PI3K: phosphatidylinositol 3-kinases
SAC: serous adenocarcinoma
TC: paclitaxel plus carboplatin
VEGF: vascular endothelial growth factor

## References

[1] Siegel R, Naishadham D, Jemal A (2012). Cancer statistics, 2012. CA Cancer J Clin.

[2] del Carmen MG, Birrer M, Schorge JO (2012). Clear cell carcinoma of the ovary: a review of the literature. Gynecol Oncol.

[3] Takano M, Tsuda H, Sugiyama T (2012). Clear cell carcinoma of the ovary: is there a role of histology-specific treatment?. J Exp Clin Cancer Res..

[4] Ozols RF, Bundy BN, Greer BE, Fowler JM, Clarke-Pearson D, Burger RA (2003). Phase III trial of carboplatin and paclitaxel compared with cisplatin and paclitaxel in patients with optimally resected stage III ovarian cancer: a Gynecologic Oncology Group study. J Clin Oncol.

[5] Itamochi H, Kigawa J, Sugiyama T, Kikuchi Y, Suzuki M, Terakawa N (2002). Low proliferation activity may be associated with chemoresistance in clear cell carcinoma of the ovary. Obstet Gynecol.

[6] Enomoto T, Kuragaki C, Yamasaki M, Sugita N, Otsuki Y, Ikegami H (2003). Is clear cell carcinoma and mucinous carcinoma of the ovary sensitive to combination chemotherapy with paclitaxel and carboplatin?. Proc Am Soc Clin Oncol.

[7] Anglesio MS, Carey MS, Kobel M, Mackay H, Huntsman DG, Vancouver Ovarian Clear Cell Symposium S (2011). Clear cell carcinoma of the ovary: a report from the first Ovarian Clear Cell Symposium, June 24th, 2010. Gynecol Oncol.

[8] Sugiyama T, Kamura T, Kigawa J, Terakawa N, Kikuchi Y, Kita T (2000). Clinical characteristics of clear cell carcinoma of the ovary: a distinct histologic type with poor prognosis and resistance to platinum-based chemotherapy. Cancer.

[9] Winter WE, Maxwell GL, Tian C, Carlson JW, Ozols RF, Rose PG (2007). Prognostic factors for stage III epithelial ovarian cancer: a Gynecologic Oncology Group Study. J Clin Oncol.

[10] Utsunomiya H, Akahira J, Tanno S, Moriya T, Toyoshima M, Niikura H (2006). Paclitaxel-platinum combination chemotherapy for advanced or recurrent ovarian clear cell adenocarcinoma: a multicenter trial. Int J Gynecol Cancer.

[11] Takano M, Kikuchi Y, Yaegashi N, Suzuki M, Tsuda H, Sagae S (2006). Adjuvant chemotherapy with irinotecan hydrochloride and cisplatin for clear cell carcinoma of the ovary. Oncol Rep.

[12] Takano M, Sugiyama T, Yaegashi N, Suzuki M, Tsuda H, Sagae S (2007). Progression-free survival and overall survival of patients with clear cell carcinoma of the ovary treated with paclitaxel-carboplatin or irinotecan-cisplatin: retrospective analysis. Int J Clin Oncol..

[13] Takakura S, Takano M, Takahashi F, Saito T, Aoki D, Inaba N (2010). Randomized phase II trial of paclitaxel plus carboplatin therapy versus irinotecan plus cisplatin therapy as first-line chemotherapy for clear cell adenocarcinoma of the ovary: a JGOG study. Int J Gynecol Cancer.

[14] Galluzzi L, Senovilla L, Vitale I, Michels J, Martins I, Kepp O (2012). Molecular mechanisms of cisplatin resistance. Oncogene.

[15] van der Schoor LW, Verkade HJ, Kuipers F, Jonker JW (2015). New insights in the biology of ABC transporters ABCC2 and ABCC3: impact on drug disposition. Expert Opin Drug Metab Toxicol..

[16] Bruhn O, Cascorbi I (2014). Polymorphisms of the drug transporters ABCB1, ABCG2, ABCC2 and ABCC3 and their impact on drug bioavailability and clinical relevance. Expert Opin Drug Metab Toxicol..

[17] O’Brien C, Cavet G, Pandita A, Hu X, Haydu L, Mohan S (2008). Functional genomics identifies ABCC3 as a mediator of taxane resistance in HER2-amplified breast cancer. Cancer Res.

[18] Wu X, Zhi X, Ji M, Wang Q, Li Y, Xie J (2015). Midkine as a potential diagnostic marker in epithelial ovarian cancer for cisplatin/paclitaxel combination clinical therapy. Am J Cancer Res..

[19] Ohishi Y, Oda Y, Uchiumi T, Kobayashi H, Hirakawa T, Miyamoto S (2002). ATP-binding cassette superfamily transporter gene expression in human primary ovarian carcinoma. Clin Cancer Res.

[20] Matsuzaki S, Serada S, Morimoto A, Ueda Y, Yoshino K, Kimura T (2014). Annexin A4 is a promising therapeutic target for the treatment of platinum-resistant cancers. Expert Opin Ther Targets..

[21] Wei B, Guo C, Liu S, Sun MZ (2015). Annexin A4 and cancer. Clin Chim Acta.

[22] Kim A, Enomoto T, Serada S, Ueda Y, Takahashi T, Ripley B (2009). Enhanced expression of Annexin A4 in clear cell carcinoma of the ovary and its association with chemoresistance to carboplatin. Int J Cancer.

[23] Kim A, Serada S, Enomoto T, Naka T (2010). Targeting annexin A4 to counteract chemoresistance in clear cell carcinoma of the ovary. Expert Opin Ther Targets..

[24] Miao Y, Cai B, Liu L, Yang Y, Wan X (2009). Annexin IV is differentially expressed in clear cell carcinoma of the ovary. Int J Gynecol Cancer.

[25] Mogami T, Yokota N, Asai-Sato M, Yamada R, Koizume S, Sakuma Y (2013). Annexin A4 is involved in proliferation, chemo-resistance and migration and invasion in ovarian clear cell adenocarcinoma cells. PLoS One.

[26] Zhou JD, Yao DM, Zhang YY, Ma JC, Wen XM, Yang J (2015). GPX3 hypermethylation serves as an independent prognostic biomarker in non-M3 acute myeloid leukemia. Am J Cancer Res..

[27] Qi X, Ng KT, Lian QZ, Liu XB, Li CX, Geng W (2014). Clinical significance and therapeutic value of glutathione peroxidase 3 (GPx3) in hepatocellular carcinoma. Oncotarget..

[28] Chen B, Rao X, House MG, Nephew KP, Cullen KJ, Guo Z (2011). GPx3 promoter hypermethylation is a frequent event in human cancer and is associated with tumorigenesis and chemotherapy response. Cancer Lett.

[29] Saga Y, Ohwada M, Suzuki M, Konno R, Kigawa J, Ueno S (2008). Glutathione peroxidase 3 is a candidate mechanism of anticancer drug resistance of ovarian clear cell adenocarcinoma. Oncol Rep.

[30] Manandhar M, Boulware KS, Wood RD (2015). The ERCC1 and ERCC4 (XPF) genes and gene products. Gene.

[31] O’Grady S, Finn SP, Cuffe S, Richard DJ, O’Byrne KJ, Barr MP (2014). The role of DNA repair pathways in cisplatin resistant lung cancer. Cancer Treat Rev.

[32] Cho YB, Chung HJ, Lee WY, Choi SH, Kim HC, Yun SH (2011). Relationship between TYMS and ERCC1 mRNA expression and in vitro chemosensitivity in colorectal cancer. Anticancer Res.

[33] Liu YP, Ling Y, Qi QF, Zhang YP, Zhang CS, Zhu CT (2013). The effects of ERCC1 expression levels on the chemosensitivity of gastric cancer cells to platinum agents and survival in gastric cancer patients treated with oxaliplatin-based adjuvant chemotherapy. Oncol Lett..

[34] Moxley KM, Benbrook DM, Queimado L, Zuna RE, Thompson D, McCumber M (2013). The role of single nucleotide polymorphisms of the ERCC1 and MMS19 genes in predicting platinum-sensitivity, progression-free and overall survival in advanced epithelial ovarian cancer. Gynecol Oncol.

[35] Reed E, Yu JJ, Davies A, Gannon J, Armentrout SL (2003). Clear cell tumors have higher mRNA levels of ERCC1 and XPB than other histological types of epithelial ovarian cancer. Clin Cancer Res.

[36] Fukumori T, Kanayama HO, Raz A (2007). The role of galectin-3 in cancer drug resistance. Drug Resist Updat..

[37] Turner JG, Dawson J, Sullivan DM (2012). Nuclear export of proteins and drug resistance in cancer. Biochem Pharmacol.

[38] Mirandola L, Yu Y, Cannon MJ, Jenkins MR, Rahman RL, Nguyen DD (2014). Galectin-3 inhibition suppresses drug resistance, motility, invasion and angiogenic potential in ovarian cancer. Gynecol Oncol.

[39] Zhang H, Luo M, Liang X, Wang D, Gu X, Duan C (2014). Galectin-3 as a marker and potential therapeutic target in breast cancer. PLoS One.

[40] Kobayashi T, Shimura T, Yajima T, Kubo N, Araki K, Wada W (2011). Transient silencing of galectin-3 expression promotes both in vitro and in vivo drug-induced apoptosis of human pancreatic carcinoma cells. Clin Exp Metastasis.

[41] Fukumori T, Oka N, Takenaka Y, Nangia-Makker P, Elsamman E, Kasai T (2006). Galectin-3 regulates mitochondrial stability and antiapoptotic function in response to anticancer drug in prostate cancer. Cancer Res.

[42] Oishi T, Itamochi H, Kigawa J, Kanamori Y, Shimada M, Takahashi M (2007). Galectin-3 may contribute to Cisplatin resistance in clear cell carcinoma of the ovary. Int J Gynecol Cancer.

[43] Zhang P, Zhang P, Zhou M, Jiang H, Zhang H, Shi B (2013). Exon 4 deletion variant of epidermal growth factor receptor enhances invasiveness and cisplatin resistance in epithelial ovarian cancer. Carcinogenesis.

[44] Lynch TJ, Bell DW, Sordella R, Gurubhagavatula S, Okimoto RA, Brannigan BW (2004). Activating mutations in the epidermal growth factor receptor underlying responsiveness of non-small-cell lung cancer to gefitinib. N Engl J Med.

[45] Popat S, Wotherspoon A, Nutting CM, Gonzalez D, Nicholson AG, O’Brien M (2013). Transformation to “high grade” neuroendocrine carcinoma as an acquired drug resistance mechanism in EGFR-mutant lung adenocarcinoma. Lung Cancer.

[46] Fujimura M, Hidaka T, Saito S (2002). Selective inhibition of the epidermal growth factor receptor by ZD1839 decreases the growth and invasion of ovarian clear cell adenocarcinoma cells. Clin Cancer Res.

[47] Ohta T, Ohmichi M, Shibuya T, Takahashi T, Tsutsumi S, Takahashi K (2012). Gefitinib (ZD1839) increases the efficacy of cisplatin in ovarian cancer cells. Cancer Biol Ther.

[48] Pedersen K, Angelini PD, Laos S, Bach-Faig A, Cunningham MP, Ferrer-Ramon C (2009). A naturally occurring HER2 carboxy-terminal fragment promotes mammary tumor growth and metastasis. Mol Cell Biol.

[49] Iqbal N, Iqbal N (2014). Human epidermal growth factor receptor 2 (HER2) in cancers: overexpression and therapeutic implications. Mol Biol Int..

[50] Eckstein N, Servan K, Girard L, Cai D, von Jonquieres G, Jaehde U (2008). Epidermal growth factor receptor pathway analysis identifies amphiregulin as a key factor for cisplatin resistance of human breast cancer cells. J Biol Chem.

[51] Guan X, Ma F, Fan Y, Zhu W, Hong R, Xu B (2015). Platinum-based chemotherapy in triple-negative breast cancer: a systematic review and meta-analysis of randomized-controlled trials. Anticancer Drugs.

[52] Tan DS, Miller RE, Kaye SB (2013). New perspectives on molecular targeted therapy in ovarian clear cell carcinoma. Br J Cancer.

[53] Kobayashi H, Kajiwara H, Kanayama S, Yamada Y, Furukawa N, Noguchi T (2009). Molecular pathogenesis of endometriosis-associated clear cell carcinoma of the ovary (review). Oncol Rep.

[54] Matsuzaki S, Enomoto T, Serada S, Yoshino K, Nagamori S, Morimoto A (2014). Annexin A4-conferred platinum resistance is mediated by the copper transporter ATP7A. Int J Cancer.

[55] Morimoto A, Serada S, Enomoto T, Kim A, Matsuzaki S, Takahashi T (2014). Annexin A4 induces platinum resistance in a chloride-and calcium-dependent manner. Oncotarget..

[56] Itamochi H, Kigawa J, Akeshima R, Sato S, Kamazawa S, Takahashi M (2002). Mechanisms of cisplatin resistance in clear cell carcinoma of the ovary. Oncology..

[57] Schwartz DR, Kardia SL, Shedden KA, Kuick R, Michailidis G, Taylor JM (2002). Gene expression in ovarian cancer reflects both morphology and biological behavior, distinguishing clear cell from other poor-prognosis ovarian carcinomas. Cancer Res.

[58] Shuck SC, Short EA, Turchi JJ (2008). Eukaryotic nucleotide excision repair: from understanding mechanisms to influencing biology. Cell Res.

[59] Huang RY, Chen GB, Matsumura N, Lai HC, Mori S, Li J (2012). Histotype-specific copy-number alterations in ovarian cancer. BMC Med Genom.

[60] Okamoto A, Sehouli J, Yanaihara N, Hirata Y, Braicu I, Kim BG (2015). Somatic copy number alterations associated with Japanese or endometriosis in ovarian clear cell adenocarcinoma. PLoS One.

[61] Fujimura M, Katsumata N, Tsuda H, Uchi N, Miyazaki S, Hidaka T (2002). HER2 is frequently over-expressed in ovarian clear cell adenocarcinoma: possible novel treatment modality using recombinant monoclonal antibody against HER2, trastuzumab. Jpn J Cancer Res.

[62] Jones S, Wang TL, Shih Ie M, Mao TL, Nakayama K, Roden R (2010). Frequent mutations of chromatin remodeling gene ARID1A in ovarian clear cell carcinoma. Science.

[63] Mabuchi S, Kawase C, Altomare DA, Morishige K, Sawada K, Hayashi M (2009). mTOR is a promising therapeutic target both in cisplatin-sensitive and cisplatin-resistant clear cell carcinoma of the ovary. Clin Cancer Res.

[64] Kato N, Sasou S, Motoyama T (2006). Expression of hepatocyte nuclear factor-1beta (HNF-1beta) in clear cell tumors and endometriosis of the ovary. Mod Pathol.

[65] Rahman MT, Nakayama K, Rahman M, Nakayama N, Ishikawa M, Katagiri A (2012). Prognostic and therapeutic impact of the chromosome 20q13.2 ZNF217 locus amplification in ovarian clear cell carcinoma. Cancer.

[66] Mabuchi S, Kawase C, Altomare DA, Morishige K, Hayashi M, Sawada K (2010). Vascular endothelial growth factor is a promising therapeutic target for the treatment of clear cell carcinoma of the ovary. Mol Cancer Ther.

[67] Yamamoto S, Tsuda H, Miyai K, Takano M, Tamai S, Matsubara O (2011). Gene amplification and protein overexpression of MET are common events in ovarian clear-cell adenocarcinoma: their roles in tumor progression and prognostication of the patient. Mod Pathol.

[68] Tan DS, Iravani M, McCluggage WG, Lambros MB, Milanezi F, Mackay A (2011). Genomic analysis reveals the molecular heterogeneity of ovarian clear cell carcinomas. Clin Cancer Res.

[69] Tan DS, Lambros MB, Rayter S, Natrajan R, Vatcheva R, Gao Q (2009). PPM1D is a potential therapeutic target in ovarian clear cell carcinomas. Clin Cancer Res.

[70] Mao TL, Shih Ie M. The roles of ARID1A in gynecologic cancer. J Gynecol Oncol. 2013;24:376–81. 10.3802/jgo.2013.24.4.376 PMC3805919 24167674

[71] Shigetomi H, Higashiura Y, Kajihara H, Kobayashi H (2012). Targeted molecular therapies for ovarian cancer: an update and future perspectives (review). Oncol Rep.

[72] Yamada Y, Shigetomi H, Onogi A, Haruta S, Kawaguchi R, Yoshida S (2011). Redox-active iron-induced oxidative stress in the pathogenesis of clear cell carcinoma of the ovary. Int J Gynecol Cancer.

[73] Aris A (2010). Endometriosis-associated ovarian cancer: a ten-year cohort study of women living in the Estrie Region of Quebec, Canada. J Ovarian Res..

[74] Kobayashi H, Yamada Y, Kanayama S, Furukawa N, Noguchi T, Haruta S (2009). The role of hepatocyte nuclear factor-1beta in the pathogenesis of clear cell carcinoma of the ovary. Int J Gynecol Cancer.

[75] Itamochi H, Kigawa J, Sultana H, Iba T, Akeshima R, Kamazawa S (2002). Sensitivity to anticancer agents and resistance mechanisms in clear cell carcinoma of the ovary. Jpn J Cancer Res.

[76] Leitao MM, Soslow RA, Baergen RN, Olvera N, Arroyo C, Boyd J (2004). Mutation and expression of the TP53 gene in early stage epithelial ovarian carcinoma. Gynecol Oncol.

[77] Alsop K, Fereday S, Meldrum C, deFazio A, Emmanuel C, George J (2012). BRCA mutation frequency and patterns of treatment response in BRCA mutation-positive women with ovarian cancer: a report from the Australian Ovarian Cancer Study Group. J Clin Oncol.

[78] Gui T, Shen K (2012). The epidermal growth factor receptor as a therapeutic target in epithelial ovarian cancer. Cancer Epidemiol..

[79] Bookman MA, Darcy KM, Clarke-Pearson D, Boothby RA, Horowitz IR (2003). Evaluation of monoclonal humanized anti-HER2 antibody, trastuzumab, in patients with recurrent or refractory ovarian or primary peritoneal carcinoma with overexpression of HER2: a phase II trial of the Gynecologic Oncology Group. J Clin Oncol.

[80] Ray-Coquard I, Haluska P, O’Reilly S, Cottu PH, Elit L, Provencher DM, Beckmann MW, Bosserman LD, et al. A multicenter open-label phase II study of the efficacy and safety of ganitumab (AMG 479), a fully human monoclonal antibody against insulin-like growth factor type 1 receptor (IGF-1R) as second-line therapy in patients with recurrent platinum-sensitive ovarian cancer. J Clin Oncol. 2013;31(suppl; abstr 5515).

[81] Bell-McGuinn KM, Matthews CM, Ho SN, Barve M, Gilbert L, Penson RT (2011). A phase II, single-arm study of the anti-alpha5beta1 integrin antibody volociximab as monotherapy in patients with platinum-resistant advanced epithelial ovarian or primary peritoneal cancer. Gynecol Oncol.

[82] Husseinzadeh N, Husseinzadeh HD (2014). mTOR inhibitors and their clinical application in cervical, endometrial and ovarian cancers: a critical review. Gynecol Oncol.

[83] Wilkinson-Ryan I, Mutch D (2013). A review of iniparib in ovarian cancer. Expert Opin Investig Drugs.

[84] Shaw D, Clamp A, Jayson GC (2013). Angiogenesis as a target for the treatment of ovarian cancer. Curr Opin Oncol.

[85] Jin Y, Li Y, Pan L (2014). The target therapy of ovarian clear cell carcinoma. Oncol Targets Ther.

[86] Rauh-Hain JA, Penson RT (2008). Potential benefit of Sunitinib in recurrent and refractory ovarian clear cell adenocarcinoma. Int J Gynecol Cancer.

[87] Rahman M, Nakayama K, Rahman MT, Nakayama N, Ishikawa M, Katagiri A (2012). Clinicopathologic and biological analysis of PIK3CA mutation in ovarian clear cell carcinoma. Hum Pathol.

[88] Miyazawa M, Yasuda M, Fujita M, Kajiwara H, Hirabayashi K, Takekoshi S (2009). Therapeutic strategy targeting the mTOR-HIF-1alpha-VEGF pathway in ovarian clear cell adenocarcinoma. Pathol Int.

[89] Lokman NA, Elder AS, Ween MP, Pyragius CE, Hoffmann P, Oehler MK (2013). Annexin A2 is regulated by ovarian cancer-peritoneal cell interactions and promotes metastasis. Oncotarget..

[90] Yamamoto S, Tsuda H, Takano M, Tamai S, Matsubara O. Loss of ARID1A protein expression occurs as an early event in ovarian clear-cell carcinoma development and frequently coexists with PIK3CA mutations. Mod Pathol Off J US Canad Acad Pathol, Inc. 2012;25:615–24. 10.1038/modpathol.2011.189 22157930

[91] Guan B, Rahmanto YS, Wu RC, Wang Y, Wang Z, Wang TL, et al. Roles of deletion of Arid1a, a tumor suppressor, in mouse ovarian tumorigenesis. J Natl Cancer Inst. 2014;106. doi:10.1093/jnci/dju146. 10.1093/jnci/dju146 PMC4056776 24899687

[92] Chandler RL, Damrauer JS, Raab JR, Schisler JC, Wilkerson MD, Didion JP (2015). Coexistent ARID1A-PIK3CA mutations promote ovarian clear-cell tumorigenesis through pro-tumorigenic inflammatory cytokine signalling. Nat Commun..

[93] Bitler BG, Aird KM, Garipov A, Li H, Amatangelo M, Kossenkov AV (2015). Synthetic lethality by targeting EZH2 methyltransferase activity in ARID1A-mutated cancers. Nat Med.

[94] Kajihara H, Yamada Y, Kanayama S, Furukawa N, Noguchi T, Haruta S (2010). Clear cell carcinoma of the ovary: potential pathogenic mechanisms (review). Oncol Rep.

[95] Senkel S, Lucas B, Klein-Hitpass L, Ryffel GU (2005). Identification of target genes of the transcription factor HNF1beta and HNF1alpha in a human embryonic kidney cell line. Biochim Biophys Acta.

[96] Littlepage LE, Adler AS, Kouros-Mehr H, Huang G, Chou J, Krig SR (2012). The transcription factor ZNF217 is a prognostic biomarker and therapeutic target during breast cancer progression. Cancer Discov..

[97] Vendrell JA, Thollet A, Nguyen NT, Ghayad SE, Vinot S, Bieche I (2012). ZNF217 is a marker of poor prognosis in breast cancer that drives epithelial-mesenchymal transition and invasion. Cancer Res.

[98] Rahman MT, Nakayama K, Rahman M, Katagiri H, Katagiri A, Ishibashi T (2012). Gene amplification of ZNF217 located at chr20q13.2 is associated with lymph node metastasis in ovarian clear cell carcinoma. Anticancer Res.

[99] Huang HN, Lin MC, Huang WC, Chiang YC, Kuo KT (2014). Loss of ARID1A expression and its relationship with PI3K-Akt pathway alterations and ZNF217 amplification in ovarian clear cell carcinoma. Mod Pathol.

